# Endovascular Treatment of Posterior Cerebral Artery Trunk Aneurysm: The Status Quo and Dilemma

**DOI:** 10.3389/fneur.2021.746525

**Published:** 2022-01-06

**Authors:** Kun Hou, Xianli Lv, Jinlu Yu

**Affiliations:** ^1^Department of Neurosurgery, First Hospital of Jilin University, Changchun, China; ^2^Department of Neurosurgery, Beijing Tsinghua Changgung Hospital, School of Clinical Medicine, Tsinghua University, Beijing, China

**Keywords:** posterior cerebral artery, trunk, aneurysm, endovascular treatment, review

## Abstract

The posterior cerebral artery (PCA) is an important artery that can be divided into four segments (P1-4): segments P1-2 are proximal segments, and segments P3-4 are distal segments. Various aneurysms can occur along the PCA trunk. True saccular aneurysms are rare, and most PCA trunk aneurysms are dissecting. Sometimes, the PCA trunk can give rise to flow-related aneurysms in association with high-flow arteriovenous shunt diseases or moyamoya disease and internal carotid artery occlusion. Some PCA trunk aneurysms require treatment, especially ruptured or large/giant aneurysms. Recently, endovascular treatment (EVT) has become the mainstream treatment for PCA trunk aneurysms, and it mainly involves reconstructive or deconstructive techniques. Traditional EVT includes selective coiling with/without stent or balloon assistance and parent artery occlusion (PAO). For proximal aneurysms, the PCA should be preserved. For distal aneurysms, PAO can be performed. However, during EVT, preservation of the PCA must naturally be the prime objective. Recently, flow-diverting stents have been used and are a revolutionary treatment for unruptured dissecting aneurysms of the PCA trunk. Despite the associated complications, EVT remains an effective method for treating PCA trunk aneurysms and can result in a good prognosis.

## Introduction

The posterior cerebral artery (PCA) is a very important pial artery; commonly, it arises from the basilar artery (BA), and uncommonly, it arises from the posterior communicating artery (PcomA) (1, 2). Similar to other pial arteries, the PCA region is susceptible to aneurysms, accounting for 0.7–2.2% of all intracranial aneurysms and 7–15% of all vertebrobasilar aneurysms (3, 4). Of the aneurysms in the PCA region, those limited to the PCA trunk are uncommon; moreover, the management of these aneurysms is difficult and complex.

Compared with aneurysms located in other areas, PCA trunk aneurysms are more frequently dissecting, presenting with fusiform, giant (even bilateral), and even serpentine and dolichoectatic shapes (5–9). A fusiform aneurysm shape can be found in up to 25% of all PCA trunk aneurysms (10). PCA trunk aneurysms can occur in isolation or in association with brain arteriovenous malformation (BAVM), dural or pial arteriovenous fistula (DAVF or PAVF), vein of Galen aneurysmal malformation, internal carotid artery (ICA) occlusion, and moyamoya disease (MMD) (11–16).

The spontaneous disappearance of PCA trunk aneurysms due to thrombosis is rare (17). They often harbor insufficient organized thrombi and are prone to hemorrhage, and intervention is necessary, especially for ruptured aneurysms (18). PCA trunk aneurysms are challenging for surgical treatment and can cause serious complications because the PCA region harbors perforating arteries, deep venous structures and cranial nerves (19, 20). Currently, endovascular treatment (EVT) has become the mainstream option for intracranial aneurysm treatment, especially EVT involving flow-diverting stents (FDSs) (21). This choice is good for PCA trunk aneurysms, especially non-mass–compressing, non-giant aneurysms (22). Until now, the understanding of EVT for PCA trunk aneurysm treatment has been insufficient. Therefore, we performed this review.

## PCA Trunk Anatomy

Embryologically, PCA arises from the ICA, but at birth, its most frequent origin is from the BA (23, 24). When the PCA originates directly from the PcomA, it is called a fetal-type PCA, with an occurrence rate of 3 to 36% (5). In fetal-type PCA, the PCA before the PcomA can be hypoplastic or absent, and the PcomA features the same diameter as or a larger diameter than the PCA (2). The PCA trunk can exhibit aplasia, hypoplasia, duplication, fenestration and other abnormalities (25–28).

Zeal and Rhoton et al. divided the PCA into four segments: the precommunicating segment (P1), ambient segment (P2), quadrigeminal segment (P3), and calcarine segment (P4). The P2 segment is long and further divided into anterior (P2A) and posterior (P2P) parts (29, 30). In the Uz study, the P2A and P2P parts were classified as two separated segments, so P1-P5 segmentation was adopted (31). Currently, P1-P4 segmentation is popular.

**Figure 1:**
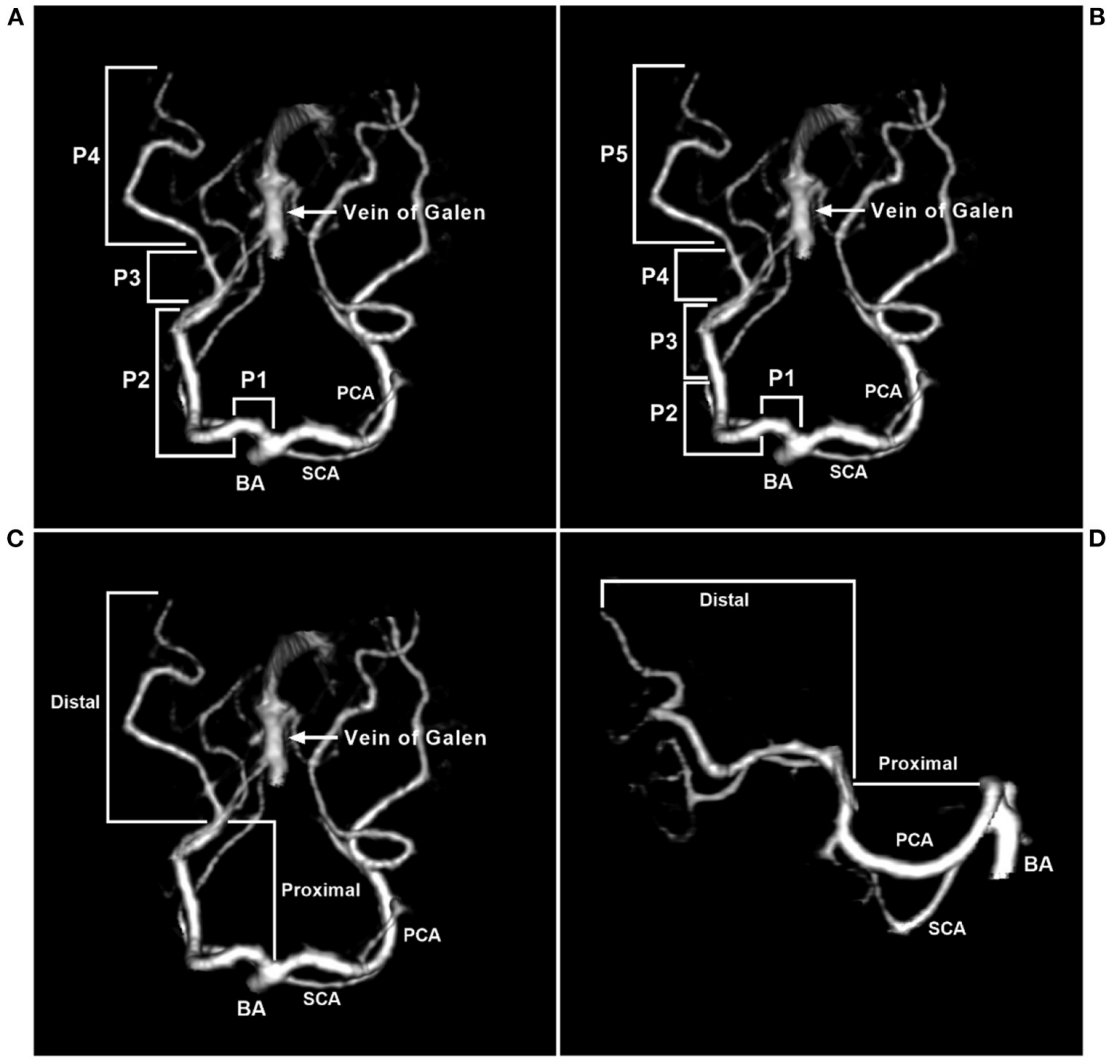
Segmentation of the PCA. **(A)** CTA of the superior-inferior view showing that the PCA was divided into four segments: P1 (precommunicating segment), P2 (ambient segment), P3 (quadrigeminal segment), and P4 (calcarine segment). P2 was further divided into anterior (P2A) and posterior (P2P) parts. **(B)** CTA of the superior-inferior view showing that the PCA was divided into five segments. **(C,D)** CTA of the superior-inferior view **(C)** and the lateral view **(D)** showing that the PCA can be divided into proximal and distal segments. BA, basilar artery; CTA, computed tomography angiography; PCA, posterior cerebellar artery; SCA, superior cerebellar artery.

The PCA can be divided into proximal and distal segments (32). P1 and P2 in the Zeal and Rhoton et al. study or P1-P3 in the Uz study belong to the proximal segment. PCA diameter and length are important for EVT. In anatomical studies, the proximal PCA mean diameter was approximately 2 mm, the distal PCA diameter at the origin was approximately 1.5 mm, and the proximal length was approximately 5 cm (31, 33). The PCA segmentations are shown in Figure 1.

The PCA gives rise to three types of branches: central perforating branches, ventricular branches (medial and lateral posterior choroidal arteries often arise from the P2 segment), and cerebral branches to the cortex and corpus callosum splenium (29, 34). In perforating branches, the P1 segment sends out important thalamoperforating arteries, short/long circumflex arteries and occasionally medial posterior choroidal arteries (35).

The PCA has anastomotic collaterals between the lateral posterior choroidal artery of the P2 segment and the anterior choroidal artery, between the long circumflex arteries of the P1 segment and the superior cerebellar artery at the level of the quadrigeminal plate, between the splenial artery of the P3-P4 segment and the posterior pericallosal artery of the anterior cerebral artery, and between the inferior temporal branch of the PCA and the superior temporal branches of the middle cerebral artery (4).

## Classifications of PCA Trunk Aneurysms

### Saccular Bifurcation or Dissecting Aneurysms

**Figure 2:**
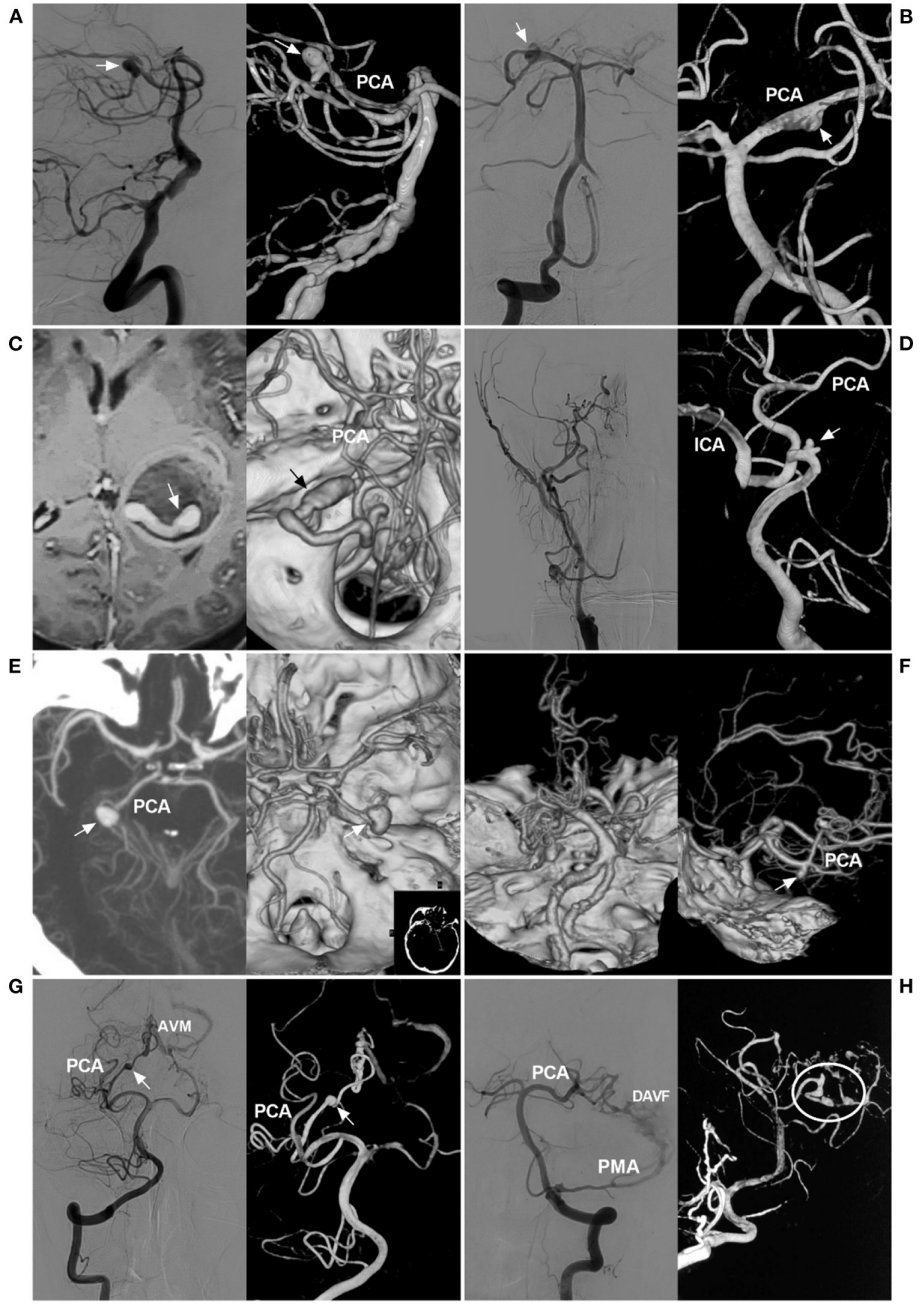
Classification of PCA trunk aneurysms. **(A)** Angiograms of the VA show a saccular true bifurcation aneurysm (arrows) at the distal segment of the PCA (left, two-dimensional angiogram; right, three-dimensional angiogram). **(B)** Angiograms of the VA show a dissecting aneurysm (arrows) at the proximal segment of the PCA (left, two-dimensional angiogram; right, three-dimensional angiogram). **(C)** MRI (left) shows an aneurysm with thrombosis [a tunnel can be seen (arrow)], and CTA (right) confirms a serpentine aneurysm (arrow) of the distal PCA. **(D)** Angiogram of the carotid artery (left) shows that the ICA was occluded at the beginning; three-dimensional angiogram of the VA (right) shows an aneurysm (arrow) located on the P1 segment, and the PCA supplies the anterior circulation. **(E)** CTA shows a P2 aneurysm (arrows) (left, maximum intensity projection; right, constructive image). **(F)** Left, CTA of the superior-inferior view shows occlusion of the bilateral middle cerebral arteries, confirming the moyamoya disease diagnosis; right, CTA of the lateral view shows an aneurysm (arrow) on the distal PCA. **(G)** Angiograms of the VA show the aneurysm (arrows) on the PCA as the feeding artery for an AVM (left, two-dimensional angiogram; right, three-dimensional angiogram). **(H)** Angiogram of the VA (left, two-dimensional DSA) shows that the PCA and PMA supply the DAVF; angiogram of the VA (right, three-dimensional angiogram) shows multiple tandem aneurysms (circle) on the PCA. AVM, arteriovenous malformation; CTA, computed tomography angiography; DAVF, dural arteriovenous fistula; ICA, internal carotid artery; MRI, magnetic resonance imaging; PCA, posterior cerebellar artery; PMA, posterior meningeal artery; VA, vertebral artery.

Saccular bifurcation aneurysms, which are true aneurysms, are rare, generally arise as a result of hemodynamic stress and result from stretching and outpouching of all of the layers of the arterial wall at the branch origin or in the fenestration of the PCA (Figure 2A) (28, 36–38). Aneurysms unrelated to the branching zone seem more likely to result from arterial dissection (Figure 2B) (38–40). Approximately 80–90% of PCA trunk aneurysms are dissecting (38, 41, 42).

PCA trunk dissecting aneurysms can be unstable, and they can grow with extension and dilatation (8). Progressive thrombosis is not uncommon in PCA trunk aneurysm growth and results in serpentine aneurysms, in which there is a twisted non-endothelialized channel of vascular course within the thrombus, accounting for 18% of all intracranial serpentine aneurysms (Figure 2C) (43, 44).

### Proximal and Distal Aneurysms

Aneurysms can be divided into proximal and distal types. Those of the P1-P2 segment were proximal aneurysms (Figures 2D,E), and those of the P3-P4 segment were distal aneurysms. This classification is important because the P1-P2 segment has important perforating arteries, and EVT must be performed more carefully (45).

For PCA trunk aneurysms, proximal aneurysms are more common (46). For instance, in a report by Ciceri et al., proximal PCA aneurysms accounted for 81% of cases (4). Specifically, in a report by Ferrante et al., PCA trunk aneurysms were localized on P1 in 22.6% of cases, the P1-P2 junction in 12% of cases, P2 in 46.7% of cases, and P3 in 18.6% of cases (6). In a report by Park et al., PCA-dissecting aneurysms involved P1 in 19.0% of cases, P1-2 junctions in 14.3% of cases, P2 in 47.7% of cases and P2-3 junctions in 9.0% of cases (38).

### Flow-Related Aneurysms

In the pathological state, PCAs can have increased blood flow, which will result in aneurysms on the PCA trunk, called flow-related aneurysms (47, 48). In occlusive diseases of the anterior circulation, such as ICA occlusion (Figure 2D), ICA agenesis or MMD (Figure 2F), increased hemodynamic stress through the PCA will lead to PCA trunk aneurysms, especially in the P1 segment, which is the collateral pathway (49–55).

Arteriovenous shunt diseases, including BAVM, DAVF, PAVF or vein of Galen aneurysmal malformations, can result in blood flow overload (Figures 2G,H) (12, 14–16, 56–60). These aneurysms are often located at the proximal PCA (61).

### Other Classifications

PCA trunk aneurysms can be divided into spontaneous (non-traumatic) or traumatic types (40, 62). Risk factors for the spontaneous type include atherosclerosis, vasculitis, inflammation/infection, connective tissue disease and genetic predisposition (63–70). Risk factors for the traumatic type include closed and open injuries because of sudden stretching or compression of the PCA against the tentorium or direct artery disruption by sharp injury, resulting in traumatic aneurysms (17, 71, 72). Traumatic aneurysms are called pseudoaneurysms with a cavity of encapsulated hematomas communicating with the lumen of the PCA (73).

**Figure 3:**
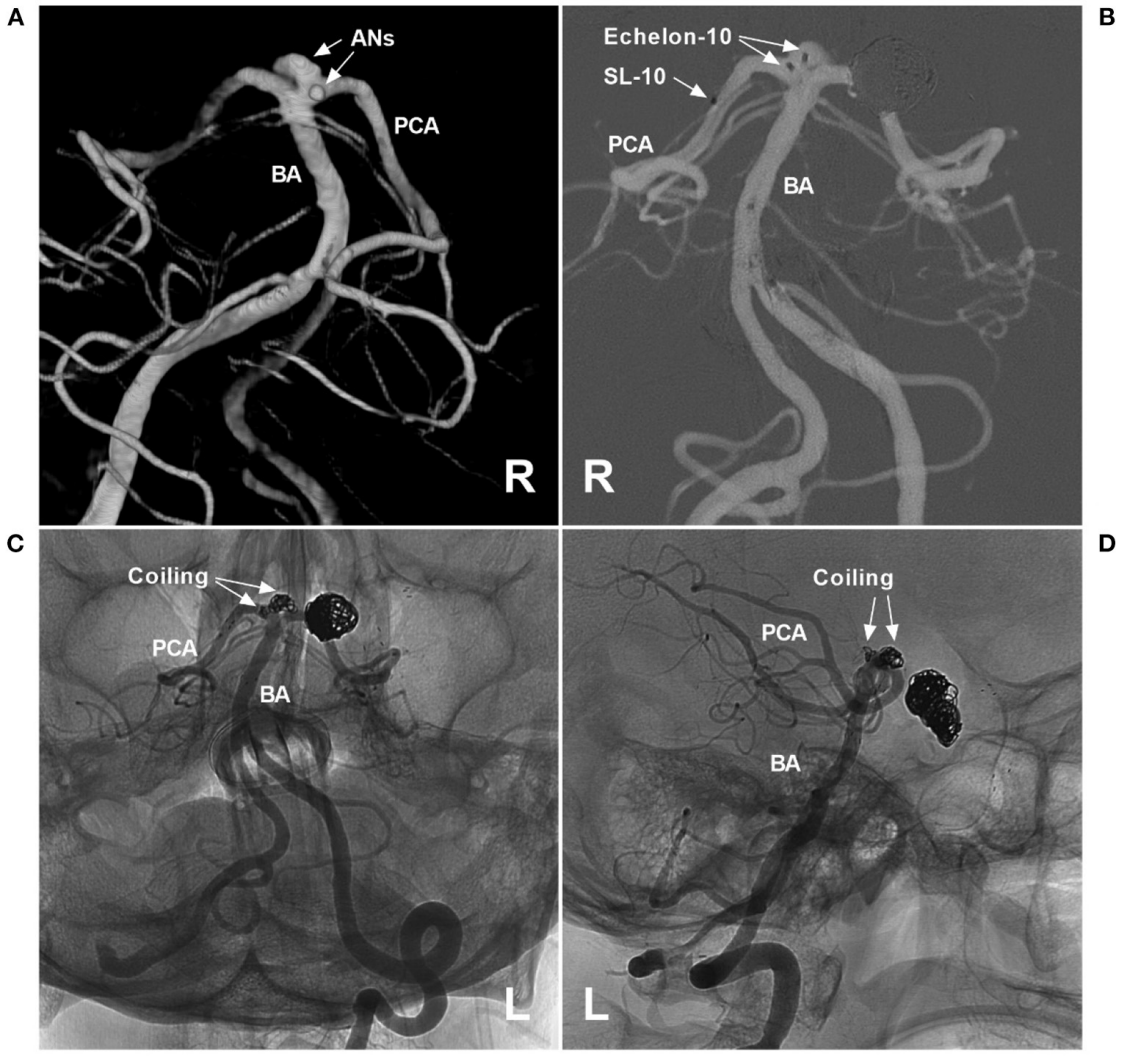
Assisted coiling for multiple P1 segment aneurysms. **(A)** Three-dimensional angiogram of the VA shows two aneurysms (arrows and ANs) on the right P1 segment. **(B)** Roadmap of the left VA shows two Echelon microcatheters (Medtronic, Irvine, CA, USA) (double arrows) that went into the aneurysms and an SL-10 microcatheter (Stryker Neurovascular, Fremont, CA, USA) (single arrow) past the aneurysms that were ready for stent deployment. **(C,D)** Unsubtracted DSA of different views shows that two aneurysms were coiled under the Atlas stent's (Stryker Neurovascular, Fremont, CA, USA) assistance. In addition, previous coiling for posterior communicating artery aneurysms can be seen. AN, aneurysm; BA, basilar artery; L, left; PCA, posterior cerebellar artery; R, right; VA, vertebral artery.

PCA trunk aneurysms can also be divided into ruptured or unruptured aneurysms; when found, half of PCA trunk aneurysms are ruptured, and most ruptured aneurysms are smaller than 10 mm and are usually distally located (4, 60, 62, 74). PCA trunk aneurysms are of various sizes; they can be divided into small, large and giant aneurysms, and large and giant aneurysms are common (3, 4, 75, 76). In a report by Ferrante et al., PCA trunk aneurysms were large in 43.6% of cases, small in 33.3% of cases, and giant in 23% of cases (6). Certainly, multiple PCA trunk aneurysms can occur (Figure 3) (10, 60, 77).

## Traditional EVT Strategies

**Figure 4:**
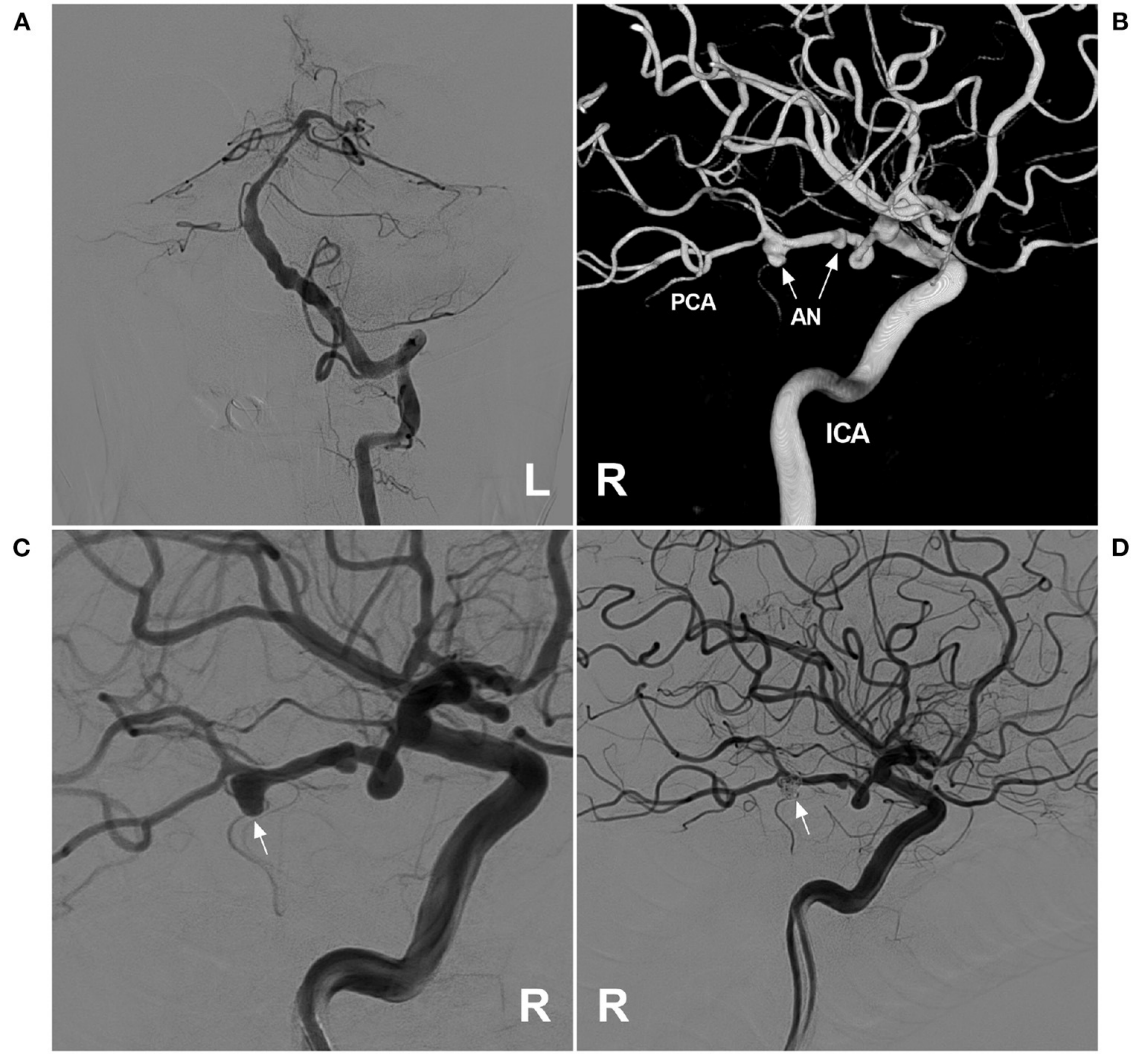
Assisted coiling for P3 segment aneurysm through the ICA. **(A)** Angiogram of the left VA showing that the BA was slim, and the right PCA was absent. **(B)** Three-dimensional angiogram of the right ICA showed two aneurysms (arrows) on the fetal-type PCA. **(C)** Angiogram of the right ICA showed that the large aneurysm (arrow) was planned to perform coiling. **(D)** Postoperative angiogram of the right ICA showed that the large aneurysm (arrow) was coiled via the ICA system. AN, aneurysm; BA, basilar artery; ICA, internal carotid artery; L, left; PCA, posterior cerebellar artery; R, right; VA, vertebral artery.

There is no standardized strategy for the management of PCA trunk aneurysms. For unruptured dissecting aneurysms, aggressive EVT is not recommended, and conservative management and anticoagulation can be chosen (68). For ruptured PCA trunk aneurysms, aggressive EVT is necessary and includes deconstructive and reconstructive methods, the choice of which depends on the aneurysm morphology and its location. However, for PCA trunk aneurysms, preservation of antegrade flow in the PCA must naturally be the prime objective. Most aneurysms of the PCA trunk are treated via the vertebrobasilar system; however, when the aneurysms are located on the fetal-type PCA trunk, or the vertebrobasilar system is occluded, the approach through the ICA is the only choice (Figure 4).

### Selective Coiling

**Figure 5:**
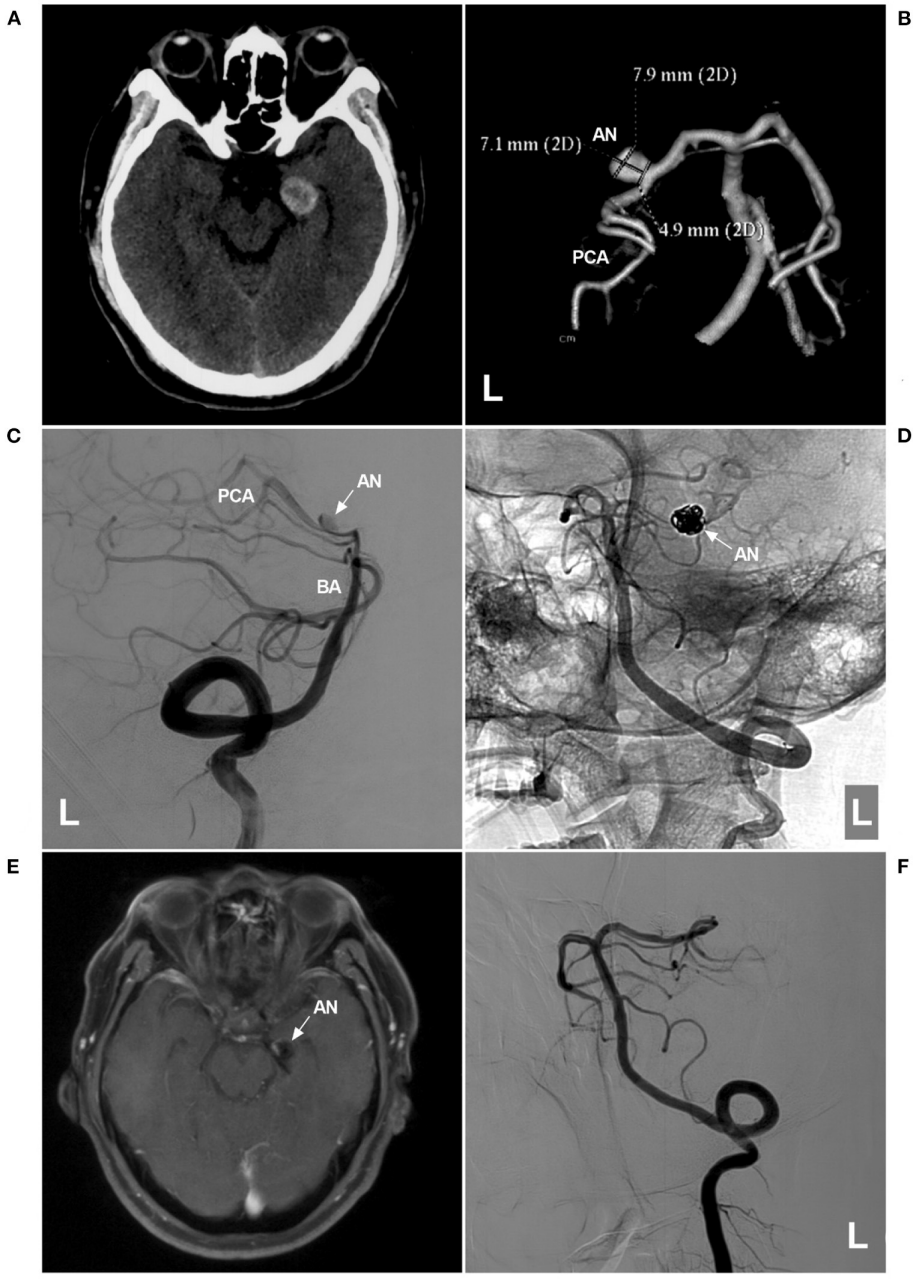
Assisted coiling of P2 segment aneurysm with thrombosis. **(A)** CT shows a highly dense lesion beside the left midbrain. **(B)** CTA showing a dissecting aneurysm (AN) on the P2 segment. **(C,D)** Two-dimensional angiograms of the left VA show the preoperative **(C)** and postoperative **(D)** images. In D, the coils (arrow in D) in the aneurysm sac were larger than the aneurysm shape in C, indicating thrombosis in the aneurysm. **(E)** One-year follow-up MRI showing that the thrombus was absorbed, and the aneurysm (arrow and AN) was smaller than that in A. **(F)** One-year follow-up angiogram showing no recurrence of the aneurysm. AN, aneurysm; BA, basilar artery; CT, computed tomography; CTA, CT angiography; L, left; MRI, magnetic resonance imaging; PCA, posterior cerebellar artery; VA, vertebral artery.

Selective coiling with/without stent or balloon assistance is an easy reconstructive method for coiling a true saccular bifurcation aneurysm while preserving the PCA at the branch origin (4, 78). In addition, in some selective cases of dissecting aneurysms, selective stent-assisted coiling with PCA preservation can be performed (Figures 3–5) (38).

### Parent Artery Occlusion

**Figure 6:**
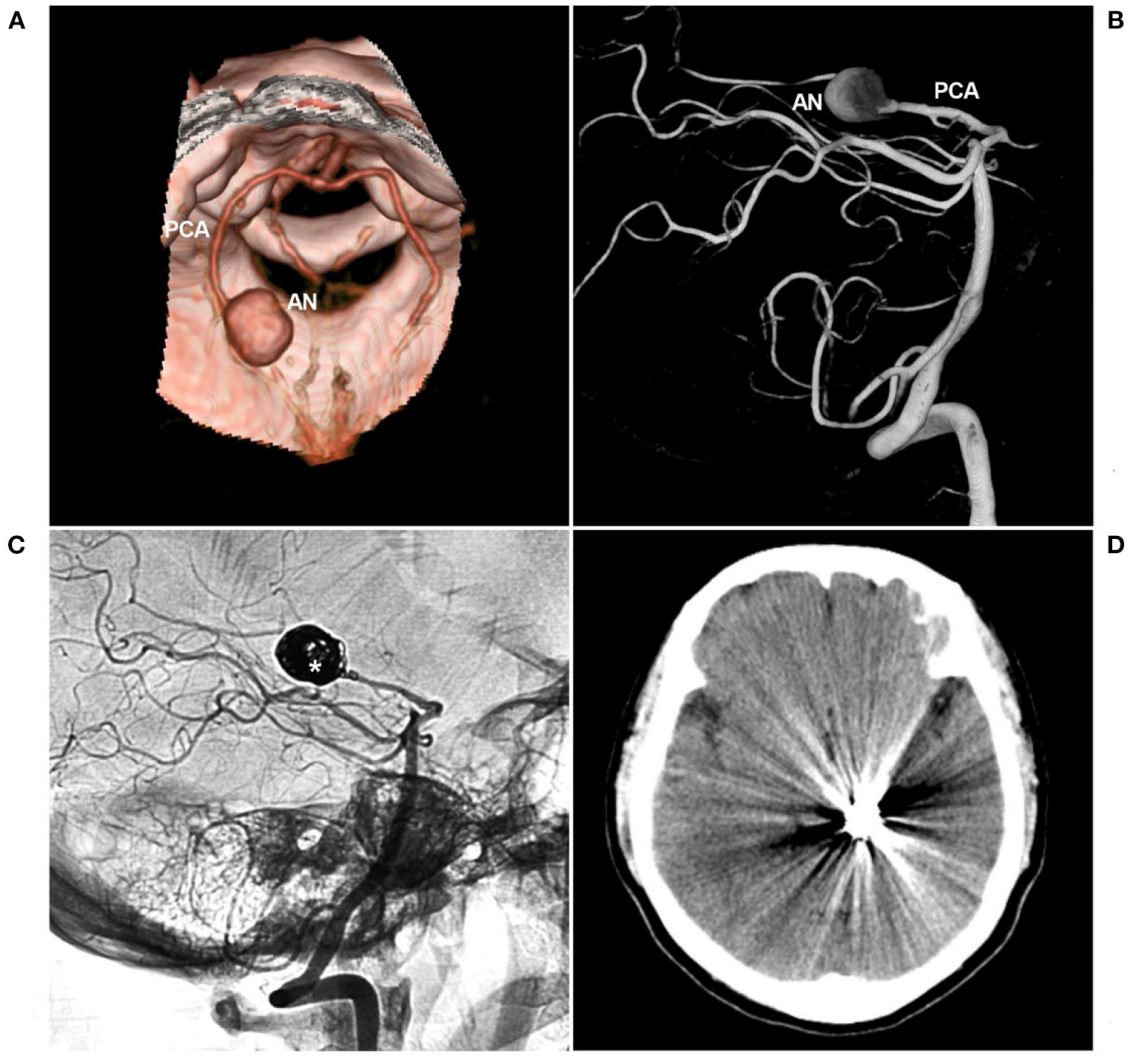
PAO for aneurysm of the P2-P3 junction. **(A,B)** CTA **(A)** and three-dimensional angiogram **(B)** showing the aneurysm (AN) on the left P2-P3 junction. **(C)** The aneurysm was treated by coiling PAO (asterisk). **(D)** Postoperative CT showing no infarction in the left occipital lobe. AN, aneurysm; CT, computed tomography; CTA, CT angiography; PAO, parent artery occlusion; PCA, posterior cerebellar artery.

Parent artery occlusion (PAO) is a deconstructive method. For some giant fusiform dissections, serpentine aneurysms, or traumatic pseudoaneurysms when PCA preservation is difficult or impossible, PAO is the last resort (Figure 6) (79, 80). PAO can be performed by coiling the proximal PCA, coiling the proximal PCA and aneurysm, or trapping the aneurysm, including the proximal and distal PCA (73).

Sometimes, aneurysm trapping is not necessary, and proximal coiling with/without aneurysm coiling is sufficient. After proximal coiling, blood-flow reversal will alter the hemodynamic stress on the aneurysm, which can produce thrombosis in the aneurysm and reduce the risk of hemorrhage (81). Only loose packing of the aneurysm or the short segment of the PCA is unreliable, resulting in coil compaction and displacement (41).

Due to the rich collateral circulation and hemodynamic balance of the PCA, PAO of the distal P3-P4 segment is safe (2, 82). However, PAO of the P1 segment should be avoided because the collateral circulation of the perforators that supply the brainstem and thalamoperforating artery are absent, and PAO will result in a serious infarct (39, 52). When PAO is the last resort, preservation of the perorating artery is mandatory, and superselective injection is useful for identifying perforating arteries arising from the proximal PCA (83).

In general, the P2 segment has well-formed anastomoses, and PAO is safe (38). However, when the PCA is fetal type, due to either an absent or a smaller P1 segment, the P2 segment can send out more perforating arteries (2). In addition, for fetal-type PCA, leptomeningeal anastomosis between the middle cerebral artery (MCA) and PCA tends to show less development of collaterals (2). Therefore, PAO of the P2 segment should be performed cautiously for fetal-type PCA (5). After PAO, steroids and low molecular weight heparin are recommended to prevent edema and sudden thrombosis of large or giant aneurysms. At the same time, it is important to maintain increased blood pressure for a few days to help the development of functional collaterals (84).

The collateral channels of the PCA region are not easily recognized. Therefore, before PAO, the balloon occlusion test (BOT) is recommended (85). The BOT should be performed when the patient is awake to allow for real-time evaluation of neurological deficits. Additionally, mean arterial pressure can be reduced to 70% of baseline during the BOT to evaluate the strength of the collateral supply to the territory at risk (22). At the same time, the BOT should be used to assess the retrograde opacification of PCA branches by collaterals (86).

In addition to the BOT, functional tests are recommended, including the superselective Wada test and provocative test (87–89). The superselective Wada test is performed through a microcatheter in the PCA under local anesthesia. After sodium amyloid injection, the patient undergoes functional examination of the PCA region (87). Under general anesthesia, a superselective provocative test can be performed with methohexital monitoring by electroencephalography or motor/somatosensory evoked potentials (88, 90).

In patients who cannot tolerate the BOT or functional tests, strong consideration should be given to EVT allowing for parent artery preservation or to neurosurgical techniques with bypass assistance (22).

### EVT for Flow-Related Aneurysms

**Figure 7:**
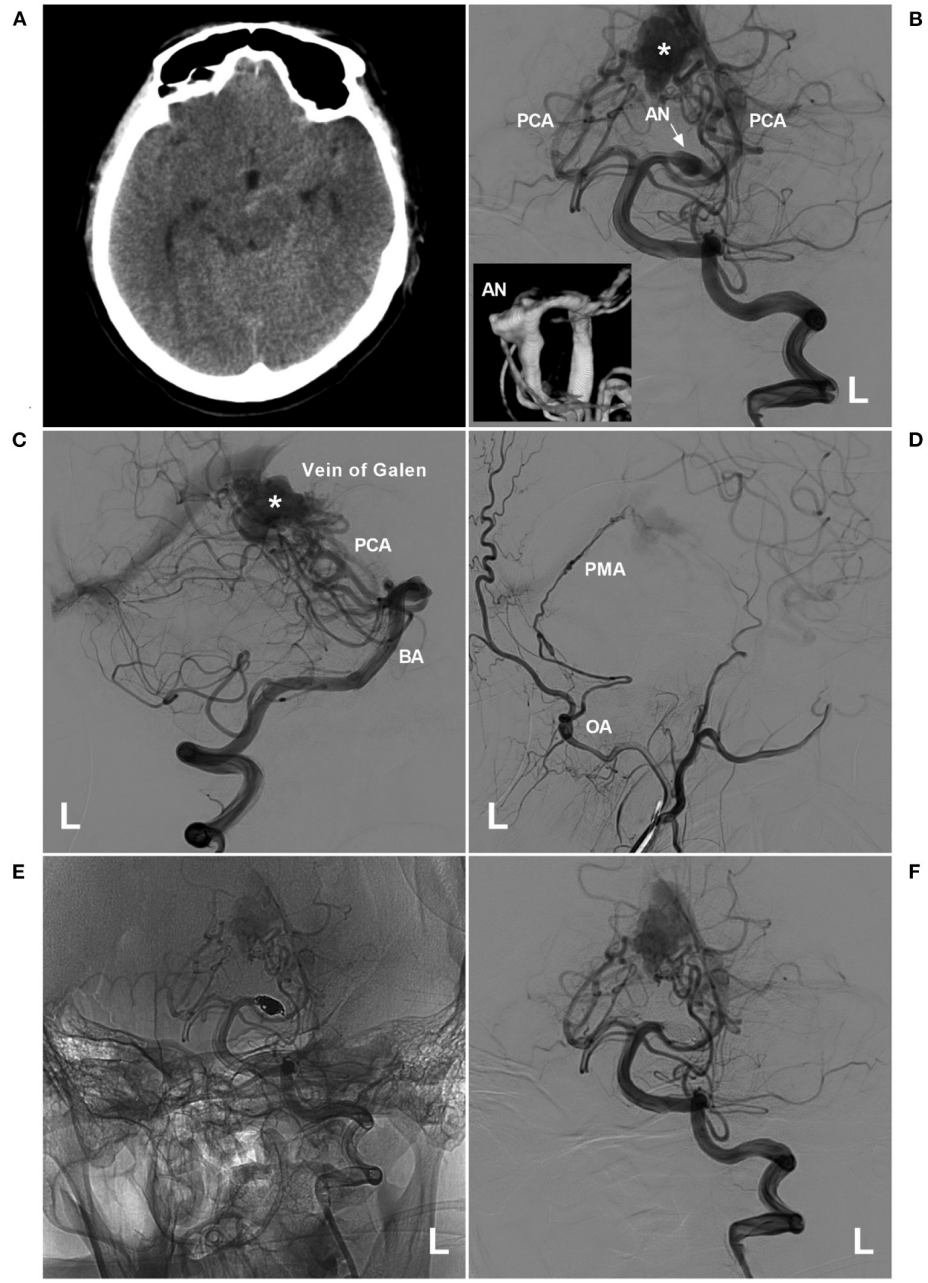
PAO for a proximal PCA aneurysm in the vein of Galen aneurysmal malformation. **(A)** Computed tomography showing a subarachnoid hemorrhage beside the left midbrain. **(B,C)** Angiograms of the left VA (B was the anteroposterior view, C was the lateral view) showing a choroidal type of vein of a Galen aneurysmal malformation, and the asterisk indicated a dilated vein of Galen; in B, an aneurysm (arrow and AN) located at the proximal PCA as the feeding artery to the vein of Galen aneurysmal malformation. In the picture inset, the aneurysm was irregular. **(D)** Angiogram of the left external carotid artery showing that the PMA from the OA also acts as the feeding artery. **(E,F)** Unsubtracted **(E)** and subtracted **(F)** angiograms of the left VA showing that the aneurysm was occluded by coiling PAO. AN, aneurysm; BA, basilar artery; L, left; OA, occipital artery; PAO, parent artery occlusion; PCA, posterior cerebellar artery; PMA, posterior meningeal artery; VA, vertebral artery.

In BAVM, DAVF, PAVF, vein of Galen aneurysmal malformations, or ICA occlusion and MMD, flow-related aneurysms of the PCA trunk abide by a similar EVT principle as isolated aneurysms (49, 53). However, for PCA trunk aneurysms located on the collateral circulation in MMD and ICA occlusion, especially P1 segment aneurysms, the PCA trunk should be reconstructed to ensure blood flow to the anterior circulation (Figure 2D) (91, 92). For distal PCA trunk aneurysms in MMD and ICA occlusion, if the collateral circulation is sufficient, PAO can be chosen (11). For flow-related aneurysms of the PCA trunk in BAVM, DAVF, PAVF or vein of Galen aneurysmal malformation cases (Figures 2G,H, 7), aggressive PAO can be considered because the PCA mainly supplies these brain arteriovenous shunts (16, 93).

Obliteration of the aneurysm prior to treating these arteriovenous shunts is preferred (12). In BAVMs, the increased pressure in the feeding artery following EVT can contribute to aneurysm rupture (94, 95). In DAVF, the pial arterial supply can be a risk factor for intraoperative hemorrhage during transarterial embolization through dural feeders (55, 96–98). Once the aneurysm is obliterated, BAVM or DAVF can be treated with any method necessary, including embolization via other feeding arteries, surgical removal or radiotherapy (74).

### Selection of Embolization Materials

For EVT with PCA preservation, coiling or stenting is the only choice (Figures 3–5). On rare occasions, a PCA trunk aneurysm can be reconstructed with parent vessel preservation using a combination of a stent and a liquid embolic agent (99). During PAO, when a PCA trunk aneurysm is isolated, coiling is preferred because the distal PCA can be preserved, which is beneficial to collateral circulation establishment (Figure 6).

For PAO of flow-related aneurysms in BAVMs or DAVFs, coiling or liquid embolization materials can be chosen. When liquid embolization material is cast, part of the material floats into the BAVM or DAVF, and the reflex occludes the aneurysm, which is safe. However, caution must be taken to avoid normal vessel occlusion due to a lack of control of the liquid embolization material (Figures 2G,H).

## Flow-Diverting STENT

In the early stages, conventional low metal coverage stents and even coronary stents were used to treat PCA trunk aneurysms (100). However, the effects of these stents are uncertain. Currently, flow-diverting stents (FDSs) are revolutionizing EVT, and off-label FDS use has extended EVT from the proximal ICA aneurysm to distal intracranial artery aneurysms, including those in the PCA trunk, especially unruptured aneurysms with no collateral or poor collateral supply (101).

**Figure 8:**
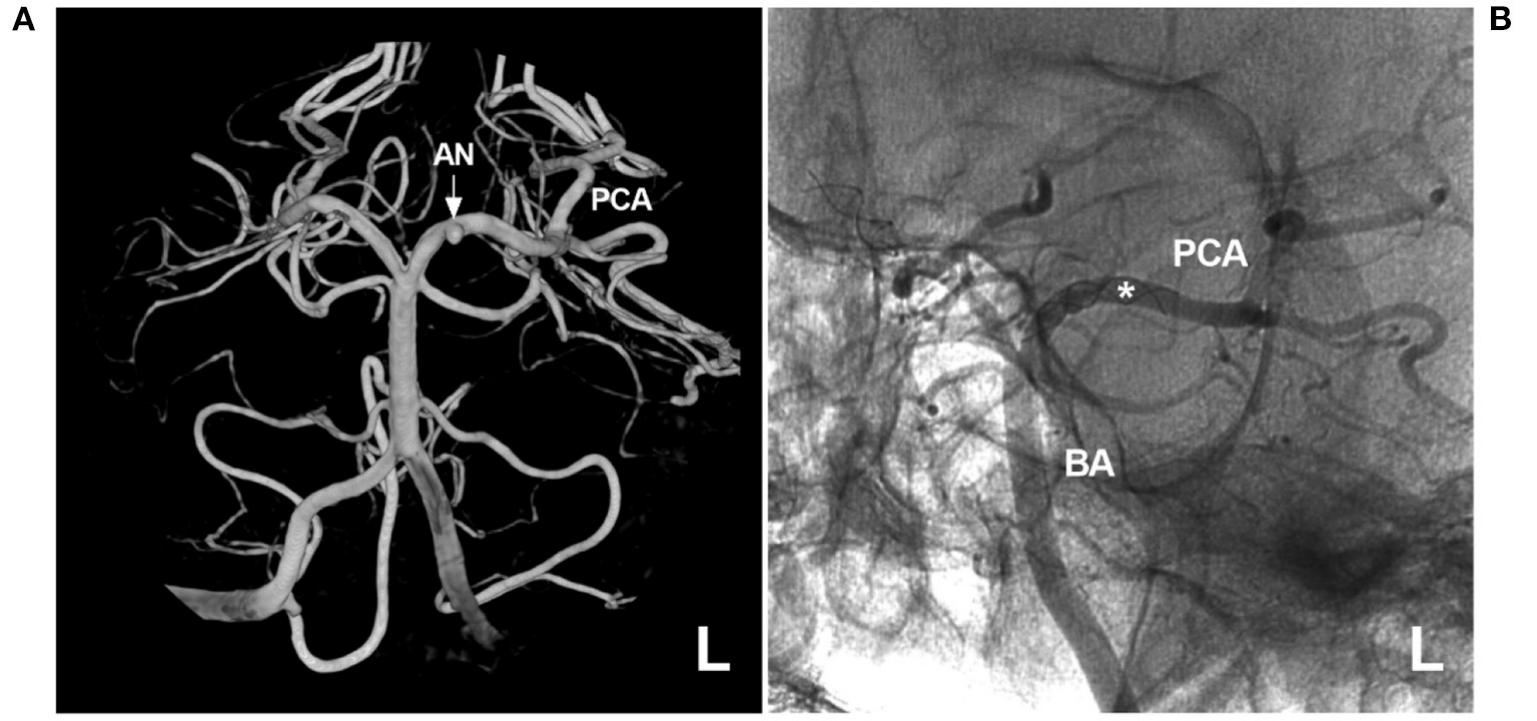
FDS deployment for a proximal PCA aneurysm. **(A)** Three-dimensional angiogram of the left VA showing a proximal PCA aneurysm (arrow and AN). **(B)** Unsubtracted angiogram showing FDS deployment (asterisk) covering the aneurysm. AN, aneurysm; BA, basilar artery; FDS, flow-diverting stent; L, left; PCA, posterior cerebellar artery; VA, vertebral artery.

**Figure 9:**
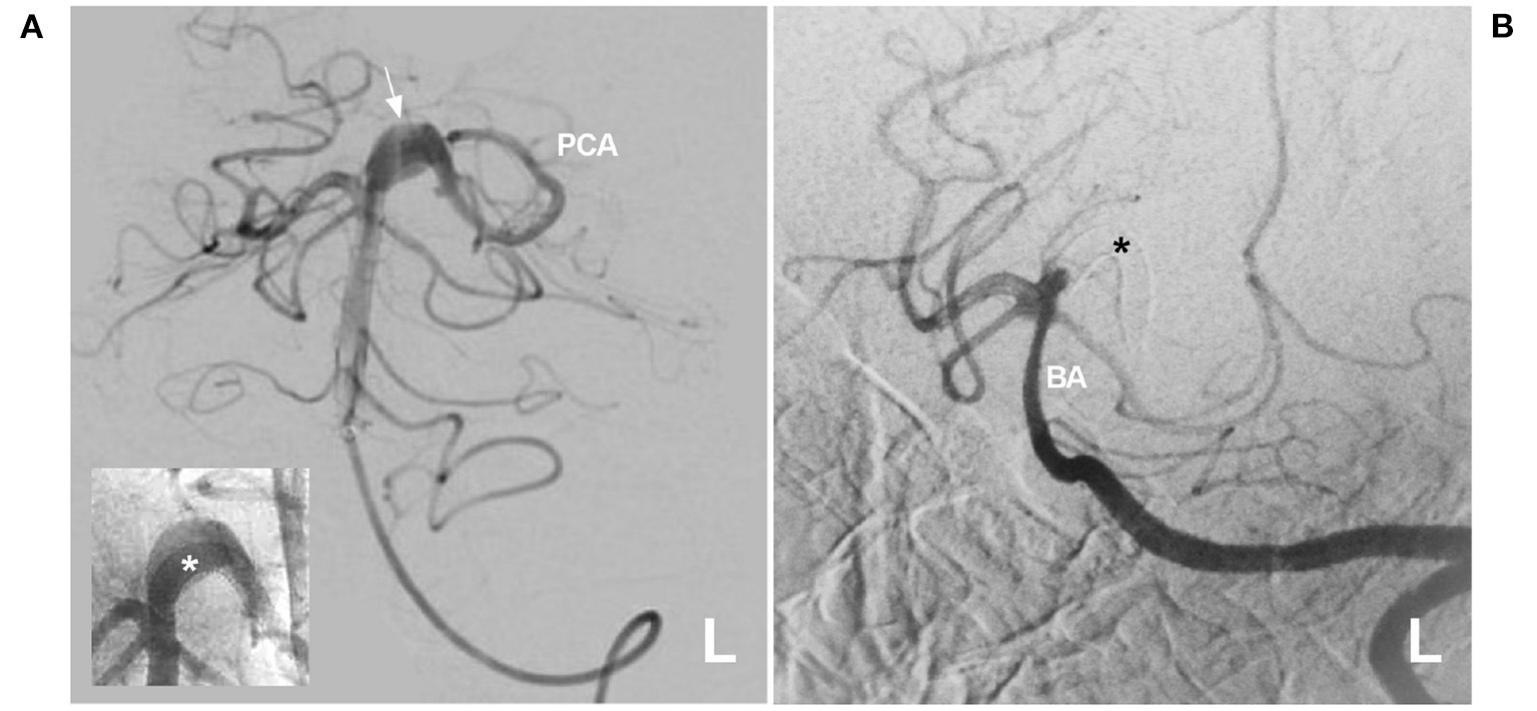
Delayed PCA occlusion after FDS deployment. **(A)** Angiogram of the left VA showing a proximal fusiform PCA aneurysm (arrow). The inset shows that the FDS (asterisk) was deployed. **(B)** Six-month follow-up angiogram showing that the proximal PCA was occluded, and the asterisk indicates the FDS location. BA, basilar artery; FDS, flow-diverting stent; L, left; PCA, posterior cerebellar artery; VA, vertebral artery.

Current FDS deployment requires a 0.027-inch microcatheter. For the proximal PCA trunk, the mean diameter is approximately 2 mm, and the length is approximately 5 cm, sufficient for FDS deployment (Figure 8) (21). However, FDS deployment in the distal vessel remains a challenge, although FDS deployment can be successful in a PCA with a tortuous path (102–104). Due to the dense perforator arteries of the P1 segment and the limitation of PCA diameter, perforating artery ischemia and varying degrees of in-stent stenosis or occlusion can occur (Figure 9) (101).

FDS application in ruptured PCA trunk aneurysms remains controversial; at this time, adjunctive coiling might be needed (101, 105, 106).

According to recent reports, FDS deployment has been used to treat flow-related aneurysms in BAVMs (107, 108). However, because the feeding arteries are dilated and thin, as in “venolization”, and moreover because the feeding artery has a greater burden of hemodynamic stress, flow-related aneurysms should be treated differently than isolated aneurysms (109). Therefore, more trials are needed to determine whether FDS deployment is feasible for PCA trunk flow-related aneurysms.

## Complications and Prognosis of EVT

**Figure 10:**
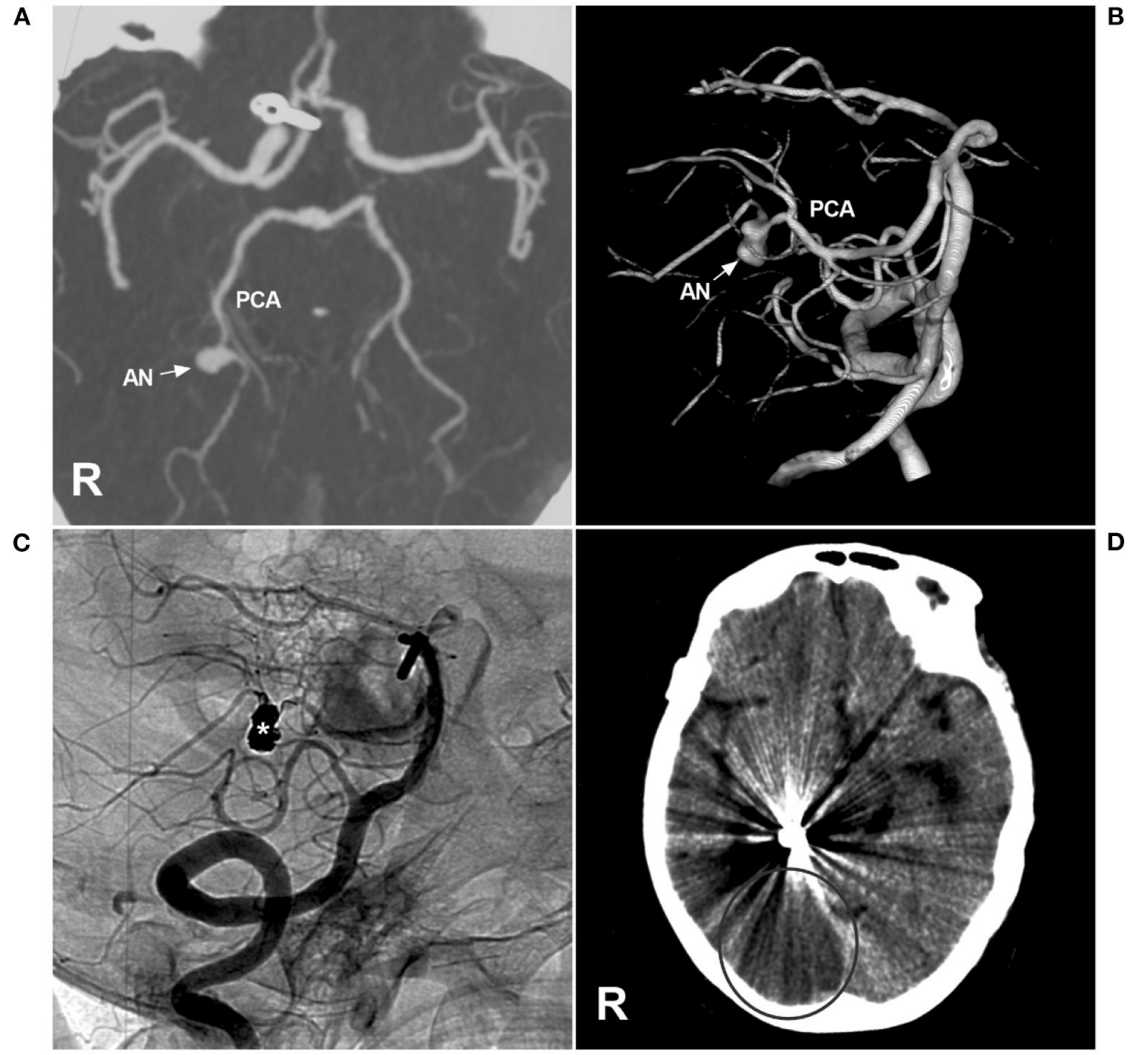
Brain infarction after PAO for P3 segment aneurysms. **(A,B)** CTA **(A)** and three-dimensional angiogram **(B)** showing the aneurysm (arrow and AN) on the right P3 segment. **(C)** The aneurysm (asterisk) was treated by coiling PAO. **(D)** Postoperative CT showing infarction in the right occipital lobe. AN, aneurysm; CT, computed tomography; CTA, CT angiography; PCA, posterior cerebellar artery; R, right; VA, vertebral artery.

Currently, EVT for PCA trunk aneurysms is effective (4). However, complications are inherent, and numerous attempts to navigate microwires and microcatheters can result in infarcts from thromboemboli or injury to arteries (101). Certainly, PAO can result in occipital infarction (Figure 10).

In 2016, Sturiale et al. reviewed 259 PCA trunk aneurysms treated with selective coiling in 37% of cases, stent-assisted coiling in 2% of cases, or PAO in 62% of cases. Immediate complete or near-complete aneurysm occlusion was achieved in 96% of cases, with an overall complication rate of 15% (105). In 2018, Wallace et al. performed a systematic review of 50 PCA trunk aneurysms treated by FDS deployment and found a complete aneurysm occlusion rate of 88% and a complication rate of 26%, and no recurrence was found (110).

Therefore, conventional EVT (especially PAO) and FDS deployment of PCA trunk aneurysms are good choices and are effective.

## Summary

The PCA is a very important artery, and aneurysms can occur along the PCA trunk. They can occur alone or as flow-related aneurysms in association with high-flow arteriovenous shunt diseases or moyamoya disease and ICA occlusion. Some aneurysms of the PCA trunk require treatment, especially ruptured or large/giant aneurysms, and EVT is a good choice. For proximal aneurysms, the PCA should be preserved; for distal aneurysms, PAO can be performed. Recently, the FDS has revolutionized the treatment of unruptured dissecting aneurysms in the PCA trunk. Despite the associated complications, EVT remains an effective method for treating PCA trunk aneurysms and can result in a good prognosis.

## Author Contributions

JY contributed to the conception, design of the manuscript, and critically revised the manuscript. KH and XL wrote the manuscript and collected the medical records of the patients. All authors approved the final version of this manuscript.

## Conflict of Interest

The authors declare that the research was conducted in the absence of any commercial or financial relationships that could be construed as a potential conflict of interest.

## Publisher's Note

All claims expressed in this article are solely those of the authors and do not necessarily represent those of their affiliated organizations, or those of the publisher, the editors and the reviewers. Any product that may be evaluated in this article, or claim that may be made by its manufacturer, is not guaranteed or endorsed by the publisher.
